# Uridine Kinase-like Protein (GhUKL4) Positively Regulates Resistance to Verticillium Wilt in Cotton

**DOI:** 10.3390/genes16070819

**Published:** 2025-07-12

**Authors:** Baimei Cheng, Yanmeng Sun, Xiaohui Sang, Jianhua Lu, Pei Zhao, Wei Chen, Yunlei Zhao, Hongmei Wang

**Affiliations:** 1 State Key Laboratory of Cotton Bio-Breeding and Integrated Utilization, Institute of Cotton Research, Chinese Academy of Agricultural Sciences, Anyang 455000, China; aachengbaimei@163.com (B.C.); sym5172@163.com (Y.S.); sangxiaohui@caas.cn (X.S.); lujianhua@caas.cn (J.L.); zhaopei@caas.cn (P.Z.); chenwei01@caas.cn (W.C.); 2 Qingfeng County Sweet Potato Industry Development Service Center, Puyang 457300, China; 3 Zhengzhou Research Base, StateKey Laboratory of Cotton Bio-Breeding and Integrated Utilization, Zhengzhou University, Zhengzhou 450000, China

**Keywords:** UKL4 uridine kinase-like protein, *Gossypium hirsutum* L., Verticillium wilt resistance

## Abstract

**Background:** Verticillium wilt (VW), caused by the fungal pathogen *Verticillium dahliae*, is a destructive disease that severely compromises cotton yield and fiber quality. Pyrimidine nucleotides, as essential metabolites and nucleic acid components, play critical roles in plant development and stress responses. However, genes involved in pyrimidine metabolism, especially their roles in disease resistance, remain largely uncharacterized in plants. **Methods:** *Ghir_D05G039120,* a gene encoding uridine kinase, shown to be associated with VW resistance in our previous study, was cloned and named as *GhUKL4*. The differential expression of *GhUKL4* between the resistant and susceptible cultivars at multiple time points post-inoculation with *V. dahliae* was analyzed by quantitative real-time PCR (qRT-PCR), and the uracil phosphoribosyl transferase (UPRT) and uridine 5′-monophosphate kinase (UMPK) domains were verified by analyzing the amino acid sequences of *GhUKL4*. The role of *GhUKL4* in the defense against VW infection was estimated by silencing *GhUKL4* in the resistant and susceptible cultivars using virus-induced gene silencing (VIGS) analysis. **Results:** There were significant differences in the expression level of *Ghir_D05G039120*/ *GhUKL4* among resistant and susceptible cotton lines. GhUKL4 contains UPRTase and UMPK domains, and there was one SNP between the resistant and susceptible cultivars in its 3′-UTR region. The silencing of *GhUKL4* reduced cotton’s resistance to VW through mediating hormone signaling (JA) and oxidative stress (ROS) pathways. **Conclusions:** GhUKL4, encoding UMPK and UPRTase domain proteins, is a new regulatory factor associated with VW resistance in *Gossypium hirsutum* through fine-tuning JA-signalling and ROS bursting.

## 1. Introduction

Nucleotides are made either by de novo synthesis from simple precursors or by so-called salvage reactions that recycle nucleobases and nucleosides released in metabolism or taken up from the environment [1]. Pyrimidine nucleotides are essential for plants. They not only act as the building blocks for nucleic acid synthesis, but also provide precursors for a wide range of cellular components, such as sugar, polysaccharides, glycoproteins, and phospholipids [2,3]. Pyrimidine nucleotides can be synthesized by de novo and salvage pathways, resulting in a common product, the nucleotide uridine 5′-monophosphate (UMP) [4]. Subsequent phosphorylation of UMP yields UDP that leads to the synthesis of all other pyrimidine nucleotides [5]. Plant pyrimidine metabolism comprises four interconnected pathways: de novo UMP synthesis, nucleotide interconversions, salvage recycling, and catabolic degradation [3]. Metabolic flux is regulated by >20 enzymes, though molecular characterization remains limited. Key examples include: *OsDHODH1* (cytosolic dihydroorotate dehydrogenase), catalyzing dihydroorotate → orotate conversion in de novo synthesis; its overexpression enhances salt/drought tolerance [6]. OsNDPK2 (nucleoside diphosphate kinase), mediating NDP → NTP phosphorylation; mutations impair chloroplast development and salt stress response [7,8].

Pyrimidine metabolism in plants involves four pathways: de novo synthesis (producing UMP), nucleotide interconversions (modifying UMP), salvaging reactions (recycling nucleosides/bases), and catabolism (degrading pyrimidines) [2,3]. Over 20 enzymes regulate these processes, but few related genes have been identified. OsDHODH1 (in de novo synthesis) converts dihydroorotate to orotate; its overexpression boosts salt/drought tolerance [6]. OsNDPK2 (in interconversion) phosphorylates NDP to NTP; its mutation disrupts chloroplast development and increases salt sensitivity [7,8]. Py-rimidine metabolism in plants also involves additional enzymes such as car-bamoylphosphate synthase, aspartate transcarbamoylase, UMP synthase, and CTP synthase, as well as nucleoside-modifying enzymes (e.g., cytidine deaminase, dihydro-pyrimidine dehydrogenase) and nucleotide hydrolases (e.g., apyrase, nucleoside tri-phosphate phosphatase) [9,10,11,12,13,14,15,16,17,18,19,20]. Genetic studies reveal that these enzymes influence diverse physiological processes, including seed germination, root development, stress adaptation, and chloroplast formation. Collectively, these findings highlight the inte-gral role of pyrimidine metabolic genes in coordinating plant growth and environmen-tal responses. Uridine kinase (UK), recycling uridine in the pyrimidine salvage pathway, is predicted to encode a dual domain (UPPTase/UMPK) that has uridine kinase at the N-terminal and UPRT at the C-terminal region [21,22]. UKLs can be classified according to their substrate specificity and source. Among them, UMPK (Uridine 5′-monophosphate kinase) is a relatively common type, whose m ain function is to phosphorylate UMP (uridine-5′-monophosphate) to generate UDP uridine diphosphate. In addition, uridine-cytidine kinase (UCK), having a broader substrate specificity, can catalyze the phosphorylation reaction of uridine and cytidine. The UK has the functional uracil phosphoribosyltransferase (UPRT), which is an important enzyme in the pyrimidine salvage pathway as it catalyzes the formation of uridine 5′-monophosphate (UMP) from uracil and phosphoribosyl-α-1-pyrophosphate (PRPP) [21,22,23].

As an important substance in pyrimidine metabolism, UK has been shown to have responses to biotic and abiotic stress in plants such as *Arabidopsis thaliana* and rice. Matchett et al. [24] found that uridine kinase is also involved in drug metabolism. Certain drugs and chemicals can be metabolized by uridine kinase and converted into corresponding phosphorylated metabolites. These metabolites may have different pharmacological activities or toxicity, affecting the efficacy and side effects of drugs [24]. Studies have shown that uridine kinase may be involved in plant immune system responses, including interactions with pathogenic microorganisms and plant resistance to pathogens. For example, Zhang et al. [25] found that the expression of uridine kinase genes in rice was significantly upregulated after infection with *Xanthomonas oryzae pv. oryzae*. This suggests that uridine kinase may play a role in rice resistance to this pathogen. However, this study did not further investigate the specific mechanism of uridine kinase involving disease resistance. In addition, in *A. thaliana*, a study showed that members of the uridine kinase gene family are involved in gene expression regulation after pathogen infection, with some being upregulated and others downregulated, implying the role of uridine kinase in the plant immune system [26]. However, the role of uridine kinase in plant disease resistance still needs further studies.

As the most economically significant species within the Gossypium genus (Malvaceae), cotton serves as the predominant source of natural textile fibers, constituting approximately 35% of global production [27,28]. Beyond its fiber applications, this crop also represents an important source of vegetable oil and animal feed. Among cultivated cotton species, G. hirsutum (upland cotton) accounts for more than 95% of worldwide production [29]. However, cotton cultivation faces significant challenges from various pathogens, particularly *V. dahliae*, the soilborne fungal pathogen responsible for VW. This vascular disease causes substantial yield losses and significantly compromises fiber quality in affected plants [30]. To resist *Verticillium* infection, cotton has evolved multiple defence mechanisms, including physiological and biochemical resistance. When pathogens infect plants, the plants themselves induce the production of antitoxins, enzymes, hormones, etc., thereby achieving the goal of resisting further pathogen infection [31,32]. For example, when plants are attacked by pathogens, the rapid production of reactive oxygen species (ROS) to inhibit pathogen growth is also a signaling molecule for early plant defense responses. Organizational structure resistance refers to the defensive effect of cotton’s own organizational structure and the induced changes in organizational structure after infection by pathogenic bacteria on *V. dahliae*. Xu et al. [33] investigated the changes in lignin deposition and content in the resistant variety Hai 7124 before and after inoculation, and found that lignin synthesis was enhanced, which plays an important role in resisting pathogen invasion. Gene regulation: Researchers have screened and cloned a large number of genes related to resistance to VW through forward or reverse genetics methods, and analyzed disease-resistant genes using VIGS, overexpression, gene editing, and protein interactions. For example, Liu et al. identified the key disease resistance gene *GausRVE2* through heterologous overexpression in cotton VIGS, *A. thaliana*, and cotton, and found that *GausRVE2* is a MYB transcription factor and a novel regulatory factor in the jasmonic acid pathway, coordinating resistance to VW [34]. Although a large number of genes related to resistance to VW have been cloned, the mechanism is still unclear.

Previous research has shown that uridine kinase is related to plant immunity, but there have been no reports on the study of uridine kinase genes in cotton. In this study, *Ghir*_*D05G039120*, a cotton homolog of the *A. thaliana* uridine kinase gene *At4G26510.1*, was identified to be responsive to *V. dahliae* infection based on QTL mapping and RNA-Seq analysis [35,36]. We conducted real-time fluorescence quantitative analysis on this gene and compared gene expression differences between resistant and susceptible materials, and named it *GhUKL4* based on the homolog of sequence cloned. Furthermore, the gene functions were estimated by silencing the gene in resistant and susceptible cotton lines and comparing the difference in resistance between silenced and unsilenced cotton plants. This study lays the foundation for an in-depth analysis of the molecular mechanism of cotton resistance to VW, and supplies the candidate gene for improving disease resistance in cotton.

## 2. Materials and Methods

### 2.1. Plant Materials and Growth Conditions

Two cotton (*Gossypium hirsutum* L.) lines—the Verticillium wilt-resistant cultivar Zhongzhimian 2 (ZZM2) and the susceptible cultivar Jimian 11 (JM11)—were selected. Seedlings of both cultivars were cultivated in plastic pots filled with solid growth medium (*v*/*v* sterile sand: vermiculite:nutritious soil = 1:1:1, Pin’S Soil, Anyang, China). Growth conditions were maintained at 25 °C (day)/20 °C (night), 60% relative humidity, and a 16/8 h light/dark photoperiod using incubators (Yuntang, Henan, China). Young leaves were harvested and stored at −80 °C for subsequent nucleic acid extraction.

### 2.2. Cloning the gDNA and cDNA of Gene GhUKL4

Genomic DNA (gDNA) and total RNA were isolated from cotton using TIANGEN Plant Genomic DNA and RNAprep Pure Plant Kits, respectively. cDNA was synthesized with HiScriptII Q Select RT SuperMix (+gDNA wiper, Vazyme, Nanjing, China). The *GhUKL4* (Ghir_D05G039120) sequences were amplified via PCR (Bio-Rad C1000, Bio-Rad, Hercules, CA, USA) under the following conditions: Primers: Designed against Ghir_D05G039120 genomic (gDNA) or mRNA (cDNA) sequences. Cycling: 94 °C/10 min; 34 cycles of 98 °C/30 s, 60 °C/30 s, 72 °C/90 s per kb; 72 °C/10 min. Reaction system 1× KOD-Plus-Neo buffer, 0.2 mM dNTPs, 1.5 mM MgSO_4_, 0.3 μM primers, 0.4 U KOD-Plus-Neo (TOYOBO, Osaka, Japan), 200 ng template. PCR products were electrophoresed, purified (FastPure Gel DNA Kit, Vazyme, Nanjing, China), cloned into pEASY-Blunt Zero vector (TransGen, Beijing, China), and sequenced (GENEWIZ, South Plainfield, NJ, USA).

### 2.3. V. dahliae Materials and Inoculation Methods

The moderately pathogenic defoliating strain *V. dahlia* Vd991 was cultured on potato dextrose agar (25 °C, 6 days). Conidia were harvested and propagated in liquid Czapek’s medium (3% sucrose, 0.2% NaNO_3_, 0.131% KH_2_PO_4_, 0.05% KCl, 0.05% MgSO_4_·7H_2_O, 0.002% FeSO_4_·7H_2_O; *w*/*v*) at 25 °C for 7 days with shaking. After filtration through four-layer gauze, conidia were quantified via hemocytometer and adjusted to 1 × 10^10^ conidia/L. Cotton seedlings at the first true-leaf stage were root-dipped in spore suspensions for 5 min. Roots, stems, and leaves were sampled at 0, 6, 12, 24, and 48 h post-inoculation (hpi), flash-frozen in liquid nitrogen, and stored at −80 °C. The 0 hpi samples served as controls for temporal expression analysis, with ≥3 biological replicates per time point.

### 2.4. Vector Construction for Virus-Induced Gene Silencing (VIGS) in Cotton and VIGS Experiments

*GhUKL4* fragments from ZZM2 and JM11 were amplified using VIGS-GhUKL4-F/R primers and cloned into pYL156 (TRV VIGS vector) via ClonExpress™ II One Step Cloning Kit (Vazyme, Nanjing, China). The positive control pYL156-GhPDS was similarly constructed. All plasmids (pYL156-GhUKL4, pYL156-GhPDS, pYL156, pYL192) were transformed into *Agrobacterium tumefaciens* GV3101 using freeze-thaw. For VIGS, bacterial suspensions were infiltrated into cotyledons of two-week-old seedlings. Experiments included ≥ 3 biological replicates with >10 plants per construct.

### 2.5. Morbidity Situation Analysis

Disease severity was quantified using a disease index (DI) based on leaf chlorosis [37]:

Grade 0: Asymptomatic

Grade 1: Symptomatic cotyledons only

Grade 2: Cotyledons + 1 true leaf affected

Grade 3: Cotyledons + 2 true leaves affected

Grade 4: Total defoliation/apical necrosis/death

$$DI=\frac{\sum \left(Number\;of\;plants\times Grade\right)}{Total\;plants\times 4}\times 100$$

### 2.6. qRT-PCR

Gene expression was analyzed by qRT-PCR using SYBR Premix Ex Taq™ II (Tli RNaseH Plus; TaKaRa, San Jose, CA, USA) in 20 μL reactions: 10 μL master mix, 2 μL cDNA, 0.8 μL each primer (10 μM), 0.4 μL ROX, 6 μL H_2_O. Reactions were run on an ABI 7500 system (95 °C/30 s; 40 cycles: 95 °C/5 s, 60 °C/34 s) with dissociation curve analysis. Cotton actin served as the internal control. Data from three biological replicates were normalized using the 2^−ΔΔCt^ method [38] and analyzed with DPS software.

### 2.7. V. dahliae Recovery

Stem segments harvested at 15 dpi from both control and TRV::*GhUKL4* plants underwent surface sterilization in 10% sodium hypochlorite (10% NaClO) for 8 min, followed by 4-5 rinses with sterile water. Using sterile surgical blades (Sangon, Shaihai, China), transverse sections (~0.8 cm depth) were excised and uniformly plated on PDA medium (Sangon, Shaihai, China) Plates were incubated at 25 °C for 5 days prior to assessment of *V. dahlia* colonization. 

### 2.8. Chemical Staining

For 3,3′-diaminobenzidine (DAB) staining, we vacuum-infiltrated the leaves with a 1 mg ml^−1^ DAB solution for one night. Subsequently, the infiltrated leaves were kept in the dark for 16 h and then destained in 95% ethanol before imaging. Nitroblue tetrazolium staining was performed as previously described [39]. The total amounts of H_2_O_2_ in root cells were determined by measuring the UV–Vis absorbance peak using tetramethylbenzidine solution incubated with GOx-NCs and glucose [40].

## 3. Results

### 3.1. Candidate Gene Differential Expression Analysis

**Figure 1:**
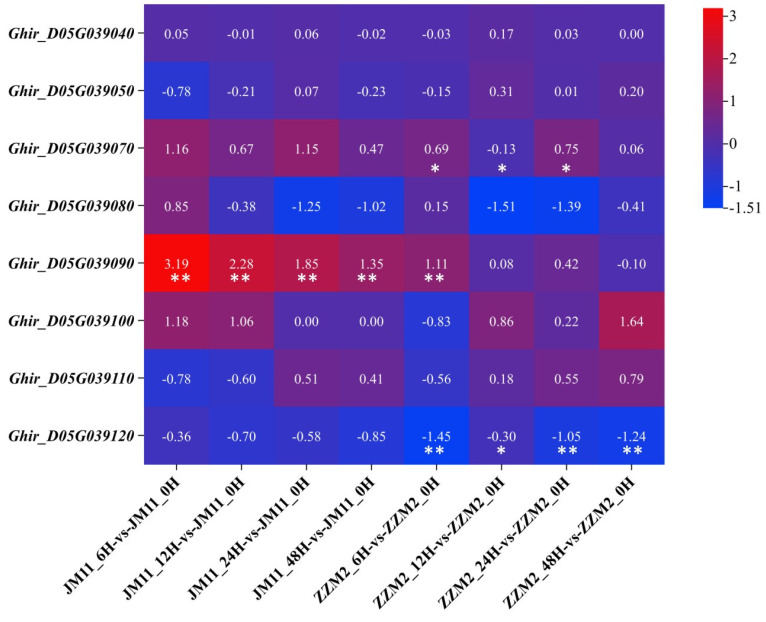
Transcriptome dynamics of *D05* cluster genes during *Verticillium* Infection. Heatmap displays log_2_(fold-change) values relative to uninfected controls. The number in each grid means log_2_(fold-change) value, and ** means significance levels of *p* < 0.001, * of *p* < 0.01. Red: upregulation; Blue: downregulation.

**Figure 2:**
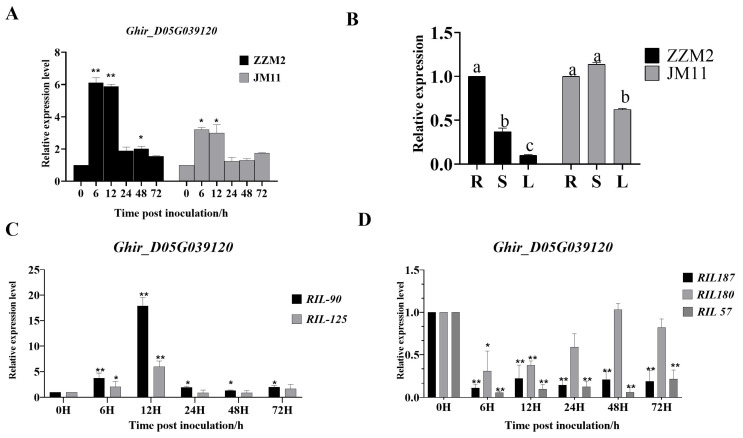
Relative expression of the target gene. (**A**): Expression levels of *Ghir_D05G039120* in V991-treated roots of cotton seedlings at different time points in ZZM2 and JM11. The expression levels were validated by RT-qPCR, significance levels: * 0.01 < *p* < 0.05, ** *p* < 0.01 (*t*-test, vs. 0 h control). (**B**): The expression patterns in different tissues for Ghir-D05G039120 homologous genes (R: root; S: steam; L: leaf). Tissue-specific profiling revealed contrasting *GhUKL4* expression patterns between cultivars (*p* < 0.05; Duncan’s test). In ZZM2, root expression (a) significantly exceeded stem (b) and leaf (c) levels. Conversely, JM11 showed equally high expression in roots and stems (a), both surpassing leaves (b). (**C**): Relative expression level of *Ghir-D05G039120* in extreme resistant cotton materials, significance levels: * 0.01 < *p* < 0.05, ** *p* < 0.01 (*t*-test, vs. 0 h control). (**D**): Relative expression level of *Ghir-D05G039120* in extreme susceptible cotton materials. significance levels: * 0.01 < *p* < 0.05, ** *p* < 0.01 (*t*-test, vs. 0 h control).

Based on the previous QTL mapping and RNA-Seq analysis [35,36], eight genes were located in the confidence interval of *qVW-D05-1* (Table 1), a major QTL on D05 chromosome, and the expression profiles of the above eight genes after infection (6, 12, 24 and 48 h after inoculation (hai)) compared to the control (0 hai) were drawn with a cut-off of *p* < 0.001 and the absolute value of log2Ratio > 1 based on the FDR < 0.0001 in RNA-Seg analysis (Figure 1). The results demonstrated that *GhUKL4* (*D05G039120*) showed significant differential expression in the resistant cultivar ZZM2, but not in the susceptible cultivar JM1l, while other genes showed no significant differential expression in the two cultivars or only in the susceptible cultivar JM11. So we chose *Ghir D05G039120* as the target gene for further study. The incidence rate of 196 cotton materials under field and greenhouse hydroponic conditions was evaluated, and selected 2 highly resistant inbred lines (RIL-90, RIL-125) and 3 highly susceptible inbred lines (RIL-180, RIL-187, RIL-57) were selected to verify the expression of *Ghir_D05G039120* (Figure S1). qRT-PCR experiments further confirmed that there were significant differences in the expression level of *Ghir_D05G039120* among eight resistant and susceptible lines including ZZM2 and JM11 after inoculation with *V. dahliae* (Figure 2A,B), In addition, we observed that the expression level of *Ghir_D05G039120* after inoculation showed an opposite trend in resistant and susceptible varieties. In resistant varieties such as ZZM2, the expression level increased 6–72 h after inoculation, while in susceptible varieties such as JM11, the expression level significantly decreased. The expression pattern of the resistant and susceptible cultivars after inoculation with *V. dahliae* was detected using qRT-PCR.

Expression levels of *Ghir_D05G039120* in different cotton tissues (roots, stems, and leaves) were analyzed in the resistant cultivar ZZM2 and the susceptible cultivar JM11 by qRT-PCR. In ZZM2, the expression level of *Ghir_D05G039120* was the highest in the root, followed by the stem, and the lowest in the leaf. In JM11, there was no significant difference in the expression level of *Ghir_D05G039120* among roots, stems, and leaves. Compared with JM11, ZZM2 showed higher expression of *Ghir_D05G039120* for the roots, but lower expression for the leaves, and no difference for the stems (Figure 2C).

### 3.2. Characterization of GhUKL4

**Figure 3:**
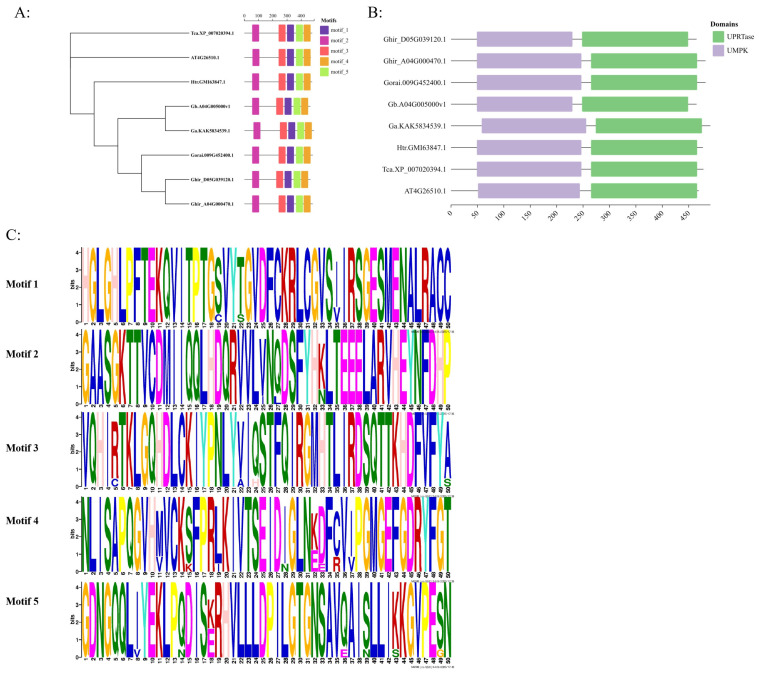

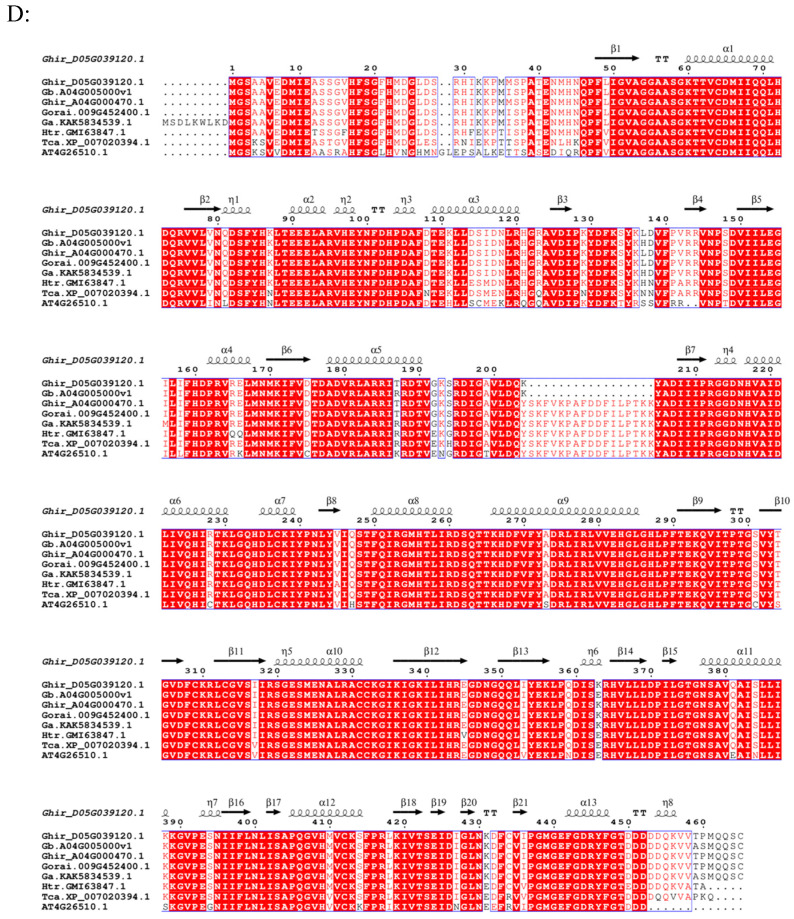

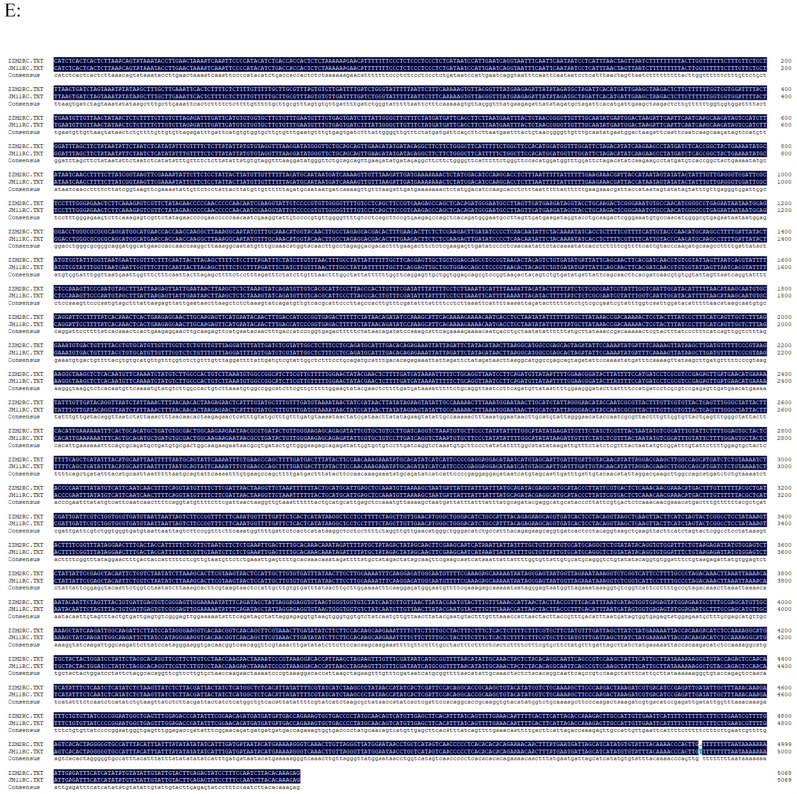

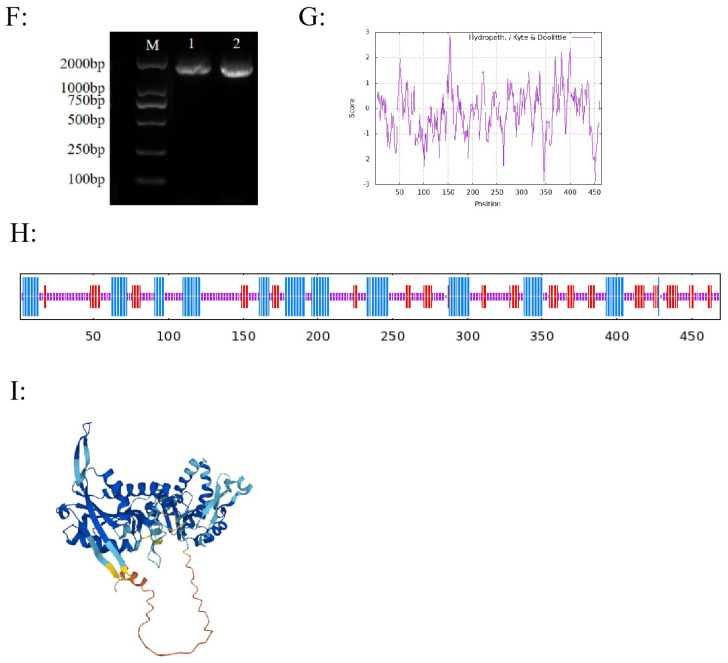
Characterization of GhUKL4. (**A**): Phylogenetic Analysis of UKL4 Proteins in Plants. Phylogenetic trees were constructed using MEGA 5.05 with the Neighbor-Joining (NJ) method. Parameters included Poisson Correction distance model and 1000 bootstrap replicates for node validation. The tree topology separates monocot and dicot clades, with *GhUKL4* clustering within the dicot lineage. (**B**): The distribution of UPRTase and UMPK in homologous genes. (**C**): Sequence Logos of Conserved Domains. WebLogo 3.7-generated sequence logos highlight conservation in UPRTase (Uracil Phosphoribosyltransferase) and UMPK (Uridylate Kinase) domains. Letter height indicates residue conservation; total stack height reflects sequence identity. (**D**): Homologous protein sequence alignment. (**E**): Comparison of the full-length sequences of *GhUKL4* from ZZM2 and JM11. *Ghir_D05G039120* was based on the annotation of the TM-1 reference genome (Sequencing version: *G.hirsutum*_ Genome_ HAU_V1.0). (**F**): Cloning length of *GhUKL4* in ZZM2 and JM11. (**G**): The hydrophilicity and hydrophobicity of GhUKL4. (**H**): Secondary Structure Prediction: SOPMA prediction of the secondary structure of GhUKL4. (**I**): Tertiary Structure Modeling:AlphaFold2-predicted GhUKL4 model.

Based on a blast search on NCBI databses, 8 proteins entified to be homologous with Ghir_D05G039120 sed on phylogenetic analysis, four proteins from Gossypium, Ghir_A04G000470.1, Gorai.009G452400.1, GbA04G005000v1 and GaKAK5834539.1, shared a high homology with Ghir_D05G039120 which is 96.47%, 96.27%, 98.71%, and 95.23%, respectively (Figure 3A). Based on analyzing sequence similarity, it was found that these homologous proteins have the same motif at similar positions (Figure 3A,B) and share the same structural domains as those of the UKL family, namely UPRTase and UMPK (Figure 3C). Through protein sequence alignment, it is found that they have similar protein secondary and tertiary structures (Figure 3D). According to the sequence information of *Ghir_D05G039120*, the 5kb-long gDNA sequence of *GhUKL4* was amplified using DNA of the resistant variety ZZM2 and the susceptible variety JM11 as templates. The sequence alignment showed that a SNP was identified in the 3′UTR of *GhUKL4* between ZZM2 and JM11 (Figure 3E); however, its functional significance requires further investigation. It was determined that the Ghir_D05G039120 belongs to the UKL family, and therefore it was named *GhUKL4*.

Using cDNA from ZZM2 and JM11 as templates, the CDS region of *GhUKL4* was cloned, and a coding region sequence of 1398 bp was obtained (Figure 3F). The protein encoded by the *GhUKL4* gene contains 465 amino acids, and there was no difference in the CDS region of *GhUKL4* between the two materials, indicating that the gene has a certain stability in inheritance in resistant and susceptible materials. The relative molecular weight of GhUKL4 is 52.17 kDa, and the theoretical isoelectric point is 6.37. The amino acids with higher content are isoleucine 9.5%, aspartic acid 8.8%, leucine 8.4%, valine 8.0%, and glycine 7.5%. Among them, there are 60 amino acid residues with negative charges and 54 amino acid residues with positive charges. The instability index of this protein is 33.31, indicating that it is a stable protein. The analysis of hydrophilicity and hydrophobicity suggests that the protein may be hydrophilic (Figure 3G). The secondary structure (Figure 3H) and tertiary structure (Figure 3I) of the GhUKL4 protein were predicted using NPSA and Swissmodel, respectively. The protein mainly contains three secondary structures: irregular coil (60.77%), alpha helix (23.45%), and main chain extension (15.78%).

### 3.3. The Silencing of GhUKL4 Reduced Cotton’s Resistance to VW

**Figure 4:**
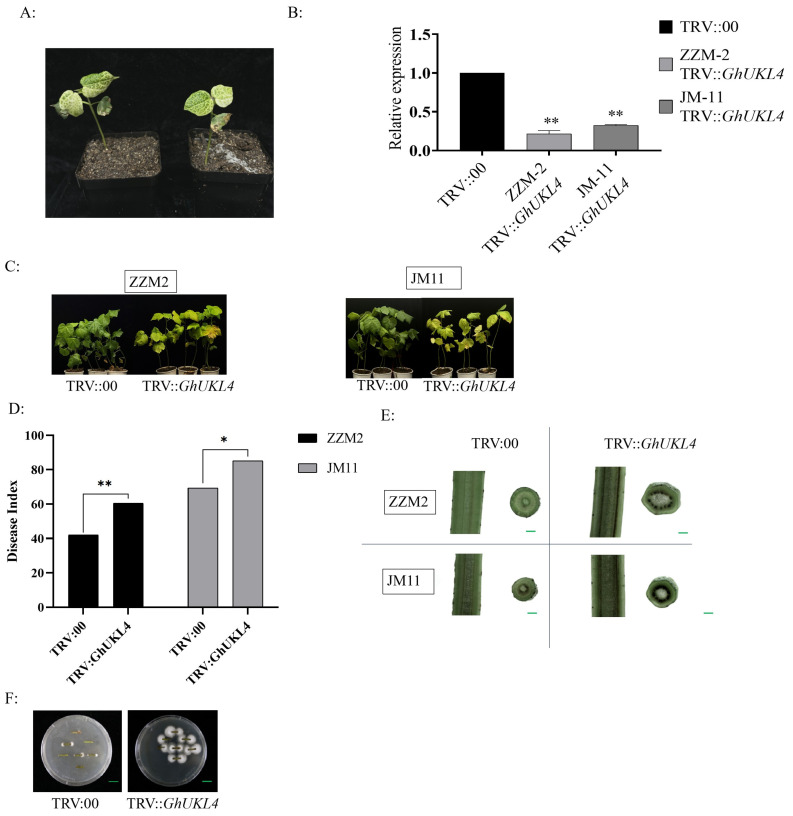
The resistance function analysis of the *GhUKL4* gene using VIGS. (**A**): Phenotypes of cotton seedlings with *GhPDS* silenced. (**B**): qRT-PCR analysis of the expression levels of *GhUKL4* in the silenced lines. “**” represent significant differences relative to each control and *p*-value < 0.01, based on Student’s *t*-test. (**C**): The phenotypes of ZZM2 and JM11 under infection with *V. dahliae* after VIGS with Agrobacterium carrying TRV::*GhUKL4* and TRV::00, and the photos were taken at 35 days after *V. dahliae* inoculation. (**D**): The disease index of plants with silenced *GhUKL4*. The qRT-PCR data were expressed relative to each control. The results were evaluated at 40 d after *V. dahliae* inoculation, with three replications containing at least 20 plants each. “*”, “**” represent significant differences relative to each control and *p* < 0.05 or *p*-value < 0.01, based on Student’s *t*-test. Each value was the mean ± SD of three biological determinations. (**E**): Vascular tissues of TRV::00 and TRV::GhUKL4 plants infected with *V. dahliae*. Scale bars = 1 cm. (**F**): Fungal recovery experiments. Scale bars = 1 cm.

To further investigate the role of *GhUKL4* in the defense against VW infection, a tobacco rattle virus (TRV)-based VIGS system was used. A *GhUKL4* fragment of about 300 bp was amplified and integrated into the pTRV2 vector to generate *GhUKL4*-knockdown cotton lines. After 10 days of VIGS injection, TRV::PDS plants showed an albino phenotype for new leaves (Figure 4A), and the relative expression level of *GhUKL4* was reduced severely in TRV::*GhUKL4* compared with the control, and silencing efficiency exceeded 80% (Figure 4B). We inoculated these plants by dipping the roots of cotton plants in Vd991 spore suspensions when the first true leaf was unfolded. After 21 days, the TRV::*GhUKL4* plants showed severe yellowing, wilting, falling leaves, and even the plants dying, while the TRV:00 plants showed mild symptoms (Figure 4C). The disease index (DI) values of TRV::*GhUKL4* plants were significantly higher than those of the TRV::00 plants after inoculation for 30 days (Figure 4D). By longitudinally and transversely dissecting the stems of TRV:*GhUKL4* and TRV::00 plants, it was found that the xylem of TRV: *GhUKL4* plants showed more brown and necrotic areas than the control (Figure 4E). The stems were also used in fungal recovery experiments to analyze the level of *V. dahliae* colonization. It was found that stems from TRV::*GhUKL4* grew more fungi than those from the control (Figure 4F), indicating that the disease resistance of cotton was significantly decreased after the *GhUKL4* gene was silenced. In conclusion, after 21 days post-inoculation (dpi), TRV::GhUKL4-JM11 plants manifested severe disease symptoms, including leaf yellowing, systemic wilting, premature defoliation, and plant mortality. Corresponding stem dissections revealed extensive and severe vascular pathogenesis, characterized by pronounced xylem browning and necrosis throughout the vascular bundles. TRV::GhUKL4-ZZM2 plants showed intermediate symptoms (mild wilting, chlorosis) and moderate vascular discoloration versus asymptomatic TRV::00-ZZM2.

### 3.4. Silencing the GhUKL4 Gene Reduces Resistance Through Multiple Pathways

**Figure 5:**
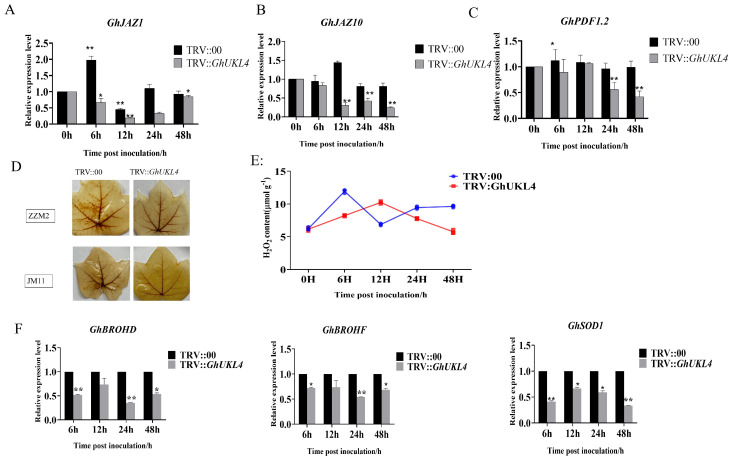
*GhUKL4* gene resistance pathway. (**A**–**C**): Relative expression of JA marker genes from ZZM2 (*GhJAZ1*, *GhJAZ10*, and *GhPDF1.2*) in TRV::*GhUKL4* compared with TRV::00 at 0, 6, 12, 24, 48 h post-inoculation with *Verticillium dahlia*, significance levels: * 0.01 < *p* < 0.05, ** *p* < 0.01 (*t*-test, vs. 0 h control) (**D**): 3,3′-Diaminobenzidine staining to indicate H_2_O_2_ accumulation in TRV: *GhUKL4* and TRV:00 at 24 h post-inoculation with *V. dahliae*. (**E**): H_2_O_2_ concentrations in the roots of ZZM2 were determined by the UV spectrophotometer method. (**F**): RT-PCR analysis of ROS pathway genes (*GhRBOHD*, *GhRBOHF*, *GhSOD1*) in the roots of control and GhUKL4-silenced plants derived from ZZM2 at 6, 12, 24, 48 hpi, significance levels: * 0.01 < *p* < 0.05, ** *p* < 0.01 (*t*-test, vs. 0 h control).

Plant hormones, such as JA, are robust signaling molecules that modulate host–pathogen interactions [41]. To explore the regulatory linkage between *GhUKL4* and defense signaling mediated JA pathways, we detected the transcript levels of related genes involved in the JA pathway in mock (TRV:00) and TRV::*GhUKL4* plants derived from ZZM2 infected by V991. Results showed that relative expression of JA signaling-related genes (*GhJAZ1*, *GhJAZ10*, and *GhPDF1.2*) was down-regulated in TRV::*GhUKL4* plants, compared with the mock (Figure 5A–C).

Considering ROS is the key player in pathogen–plant interactions [42], we conducted a comparative analysis of ROS during the early stages of *V. dahliae* invasion in *GhUKL4* silencing lines. Our results revealed that *V. dahliae* infection induced H_2_O_2_ production in control leaves, but the knockdown of *GhUKL4* expression inhibited the accumulation of H_2_O_2_ under *V. dahliae* infection (Figure 5D). Subsequently, we determined H_2_O_2_ concentrations in roots of ZZM2 with a UV-spectrophotometer. H_2_O_2_ concentrations in the control were significantly induced following *V. dahliae* invasion and had peak values at 0–12 h post-inoculation (hpi). Remarkably, silencing *GhUKL4* not only delayed the peak of H_2_O_2_ induction but also significantly decreased H_2_O_2_ accumulation during the *V. dahliae* infection (Figure 5E). These results all indicate that *GhUKL4* plays an important role in resistance to *V. dahliae* stress. To further elucidate *GhUKL4*’s role in regulating the ROS pathway, we analyzed the expression of key ROS-generating and scavenging genes via RT-PCR. We focused on the primary enzymatic sources of the defense-related oxidative burst: the NADPH oxidases *GhRBOHD* and *GhRBOHF* [43], responsible for apoplastic superoxide (O_2_^−^) production, and the cytosolic superoxide dismutase *GhSOD1*, which rapidly converts O_2_^−^ to H_2_O_2_. Consistent with the impaired H_2_O_2_ burst in silenced plants [44], RT-PCR analysis revealed that *GhUKL4* knockdown significantly downregulated the pathogen-induced expression of both *GhRBOHD* and *GhRBOHF* (Figure 5F). Moreover, *GhSOD1* expression was also reduced in silenced lines upon infection (Figure 5F).

## 4. Discussion

### GhUKL4 Is Associated with Verticillium Wilt Resistance in Cotton

In this study, we identified the *GhUKL4*, which is located in the region of a major QTL *qVW-D05-1* in our previous studies [36]. The confidence interval of *qVW-D05-1* was 1.02 cM in physical distance and contained eight genes, as shown in Table 1. Based on the transcriptome data from our previous study [35], only *GhUKL4* (*D05G039120*) showed significant differential expression after infection (6, 12, 24 and 48 h after inoculation (hai) compared to the control (0 hai) in the resistant cultivar ZZM2 (Figure 1), and the differential expression was further verified in 2 highly resistant inbred lines (RlL-90, RIL-125) and 3 highly susceptible inbred lines (RIL-180, RIL-187, RIL-57), as is shown in Figure 2, It is interesting that *GhUKL4* showed up-regulated expression after infection (6, 12, 24, 48 and 72 h after inoculation (hai) compared to the control (0 hai)in the two extremely resistant inbred lines, but down-regulated expression in the three extremely susceptible inbred lines (Figure 2). Our previous studies showed that the gNA sequence variation or expression difference of those genes involved in the basal defense in diverse cotton lines might be the molecular mechanisms of VW resistance in *G. hirsutum* [35]. A recent study showed that a natural SNP variation in the promoter of a pathogenesis-related protein gene leads to the differential expression of the target gene and plant disease resistance [45]. The gDNA sequence of *GhUKL4* contained a SNP in the 3′UTR region between ZZM2 and JM11 (Figure 3E), although the UTR region does not encode amino acids, it plays important regulatory roles in mRNA translation, stability, and intracellular localization [44]. We inferred that the SNP variation in the 3′UTR or the promoter between the resistant and susceptible lines may contribute to the expression differences of *GhUKL4* between the resistant and susceptible cultivars after inoculation with *V. dahliae*, implying the role of uridine kinase in the plant immune system.

GhUKL4 belongs to the UKL family and positively regulates Verticillium wilt in cotton.

Protein structure prediction of GhUKL4 revealed that it belongs to the UKL family and shares the same structural domains as those of the UKL family, namely UPRTase and UMPK (Figure 3C). These domains are critical for the function of UKL family proteins, which are involved in uracil metabolism and nucleotide synthesis [21,22]. It was reported that members of the uridine kinase gene family are involved in gene expression regulation after pathogen infection [26], which showed that the uridine kinase gene family contributes to disease resistance through specialized maintenance of nucleotide homeostasis and RNA quality control. Orthologs in crop species likely perform analogous “immune sanitation” functions, positioning uridine kinase family members as critical regulators of plant immunity through structural specialization in nucleotide salvage. In this study, *GhUKL4* showed significant expression differences between the resistant and the susceptible cultivar after inoculation with *V. dahliae*, and showed up-regulated expression after intection in the resistant inbred lines, but down-regulated expression in the susceptible inbred lines (Figure 2), which is in accordance with the results in previous study, showing that the expression of uridine kinase genes in rice was significantly upregulated after infection with *X. oryzae pv. oryzae* [25]. Silencing of *GhUKL4* in cotton using VIGS showed more severe yellowing and wilting (Figure 4C), which was confirmed by disease index, stem longitudinal section, and fungal recovery experiments (Figure 4D–F), This indicates that *GhUKL4* positively regulates resistance to *Verticilium* wilt in cotton, implying that *GhUKL4* plays a crucial role in cotton’s defense against *Verticillium* wilt.

*GhUKL4* exerts disease resistance through the JA pathway and ROS burst.

The differential expression (partial up-regulation/down-regulation) of uridine kinase members in *A. thaliana* following pathogen infection suggests potential involvement of this gene family in plant immune regulation, though specific mechanisms remain elusive [26]. Jasmonic acid (JA), a defensive plant hormone, synergizes or antagonizes with common hormones during plant growth to regulate developmental processes such as metabolite synthesis, pest/disease defense, and organogenesis [46]. Reactive oxygen species (ROS, e.g., H_2_O_2_) function as defense signaling molecules that directly kill pathogens, and impaired ROS production may enhance pathogen colonization capacity [47]. This study investigates the association between *GhUKL4* (a cotton uridine kinase-like gene) and disease resistance, particularly its potential role in mediating hormone signaling (JA) and oxidative stress (ROS) pathways.

After silencing *GhUKL4*, the expression of key JA pathway genes (*GhJAZ1/10*, *GhPDF1.2*) following V991 infection was significantly downregulated in silenced plants (Figure 5A–C), indicating that *GhUKL4* positively regulates the JA signaling pathway. Silenced plants exhibited drastically reduced H_2_O_2_ levels (Figure 5D). We infer that GhUKL4 may be involved in the JA defense pathway of plants, thereby enhancing their disease resistance. Additionally, *GhUKL4* might facilitate rapid ROS bursts through modulating NADPH oxidase (RBOH) activity or inhibiting antioxidant enzymes.

A potential crosstalk mechanism is hypothesized: JA may activate ROS production via RBOH genes (*GhRBOHD, GhRBOHF*) and superoxide dismutase: GhSOD1 (cytosolic O_2_^−^-to-H_2_O_2_ converter), while ROS could amplify JA signaling, forming a positive feedback loop. *GhUKL4* likely acts as a hub at this intersection, integrating hormonal and oxidative stress responses.

## 5. Conclusions

In conclusion, our study identified *GhUKL4*, encoding UMPK and UPRTase domain proteins, which is significantly associated with VW resistance in *G. hirsutum*. We concluded that UKL4, a new regulatory factor, plays a pivotal role in fine-tuning JA-signalling and ROS bursting, which would improve our understanding of the mechanisms underlying the resistance to VW.

Future studies should aim to further elucidate the precise molecular mechanisms by which *GhUKL4* regulates JA signaling and ROS production. This could involve detailed investigation of *GhUKL4* protein interactions, its subcellular localization, and its impact on the expression and activity of downstream effectors in the JA pathway and ROS production pathways. Additionally, it would be interesting to explore the potential cross-talk between JA signaling and ROS production in the context of *GhUKL4*-mediated resistance to *V. dahliae* and other pathogens. Such insights could pave the way for the development of novel strategies to enhance crop resistance against pathogenic infections.

## Figures and Tables

**Table 1:** Annotation of candidate genes located in the confidence interval *qVW-D05-1* from our previous study [36].

Gene ID	Gene Name	Description	Start	End	Strand
*Ghir_D05G039040*	*SFT2*	Protein transport protein SFT2	62,511,198	62,514,773	+
*Ghir_D05G039050*	*recX*	Regulatory protein RecX	62,526,388	62,534,437	-
*Ghir_D05G039070*	*UGT76A2*	UDP-glucose iridoid glucosyltransferase	62,535,623	62,537,817	-
*Ghir_D05G039080*	*UGT76A2*	UDP-glucose iridoid glucosyltransferase	62,540,122	62,541,578	-
*Ghir_D05G039090*	*UGT76A2*	UDP-glucose iridoid glucosyltransferase	62,549,851	62,551,307	-
*Ghir_D05G039100*	*NA*	NA	62,552,046	62,553,225	-
*Ghir_D05G039110*	*KIN14F*	Kinesin-like protein KIN-14F	62,553,683	62,560,508	-
*Ghir_D05G039120*	*UKL4*	Uridine kinase-like protein 4	62,576,759	62,581,764	+

## Data Availability

Data requirements will be provided as requested.

## Supplementary Materials

The following supporting information can be downloaded at: https://www.mdpi.com/article/10.3390/genes16070819/s1, Figure S1: The situation after extreme material vaccination with V991. Table S1: Gene Naming. Table S2: Primer Sequence.

## Abbreviations

The following abbreviations are used in this manuscript:

VW	Verticillium wilt
UMPK	Uidine 5′-monophosphate kinase
UPRT	Uacil phosphoribosyltransferase
UKL	Uridine kinase like
VIGS	Virus-Induced Gene Silencing
ROS	Reactive Oxygen Species
JA	Reactive Oxygen Species
MDPI	Multidisciplinary Digital Publishing Institute
DOAJ	Directory of open access journals
TLA	Three letter acronym
LD	Linear dichroism
