# Supplementary material for: Uridine Kinase-like Protein (GhUKL4) Positively Regulates Resistance to Verticillium Wilt in Cotton
Source: Genes (Basel). 2025 Jul 12;16(7):819. doi: 10.3390/genes16070819 (PMC12294303; doi:10.3390/genes16070819)
Supplement: Supplementary file 1 [file genes-16-00819-s001.zip › Figure S1.pdf]

Figure S1:The situation after extreme material vaccination with V991.

A.The average disease level of extreme cotton materials under hydroponic conditions.

B.The average disease level of extreme cotton materials under soil cultivation conditions.

C.Phenotype of extreme cotton material infected with V991 under hydroponic conditions.

A

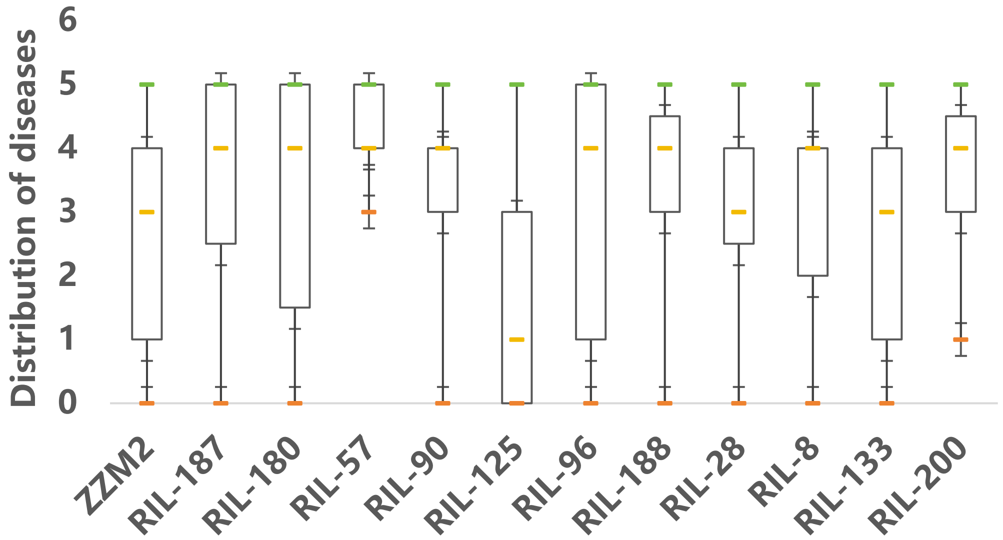

B

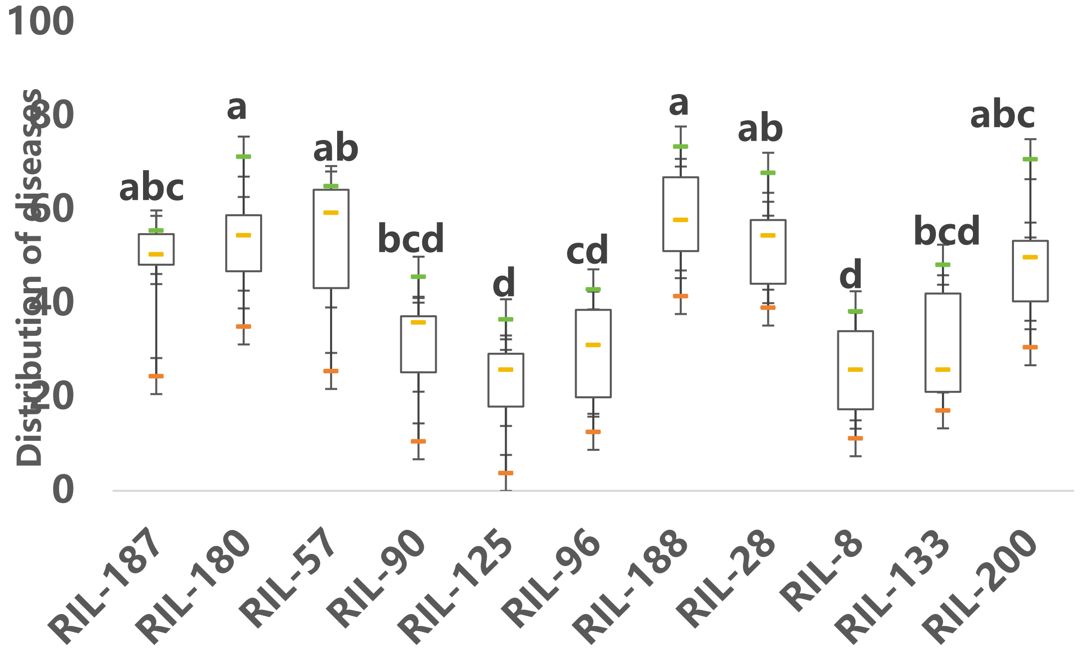

C

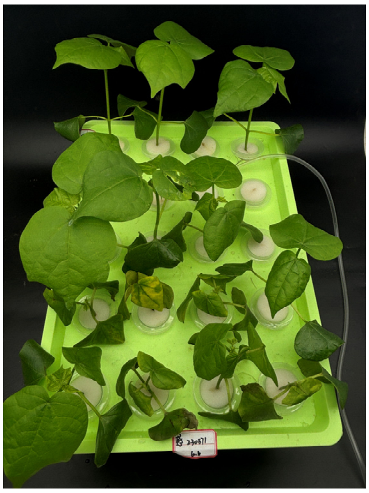

RIL-90

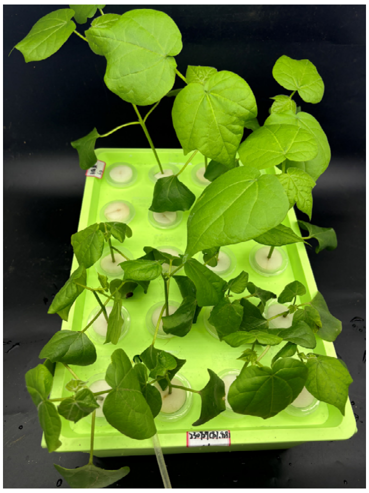

RIL-125

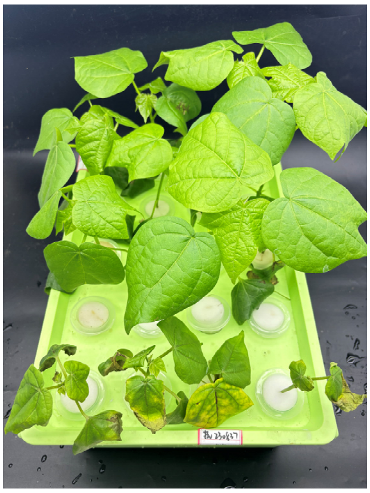

ZZM2

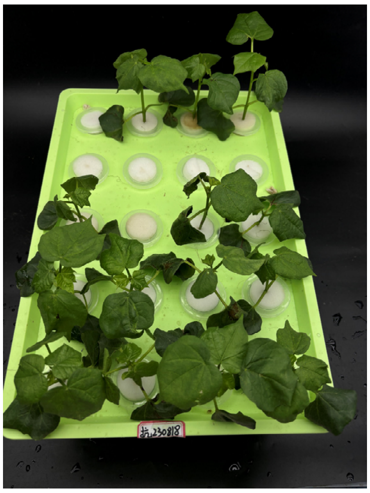

RIL-57

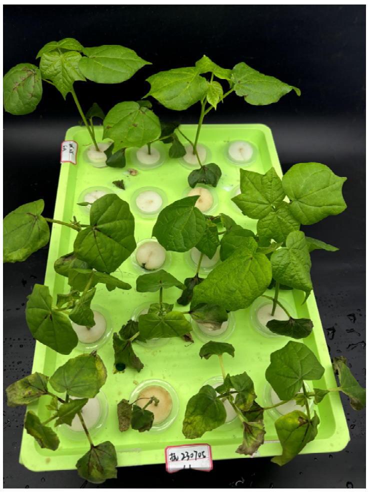

RIL-180

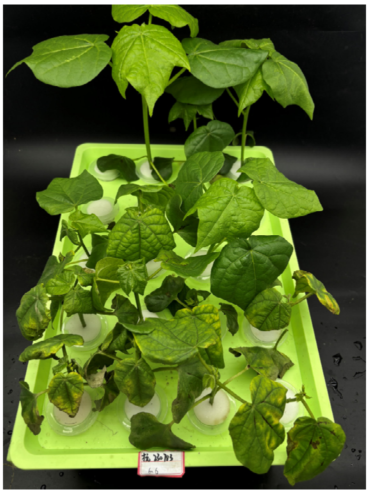

RIL-187
